# Manifestations of Rheumatic Carditis, Regression of Valvular Regurgitation, and Independent Predictors of Mitral Regurgitation Improvement After Rheumatic Carditis in Thai Children

**DOI:** 10.5334/gh.1295

**Published:** 2024-02-07

**Authors:** Araya Kaewpechsanguan, Paweena Chungsomprasong, Kritvikrom Durongpisitkul, Chodchanok Vijarnsorn, Prakul Chanthong, Supaluck Kanjanauthai, Thita Pacharapakornpong, Ploy Thammasate, Jarupim Soongswang

**Affiliations:** 1 Division of Pediatric Cardiology, Department of Pediatrics, Faculty of Medicine Siriraj Hospital, Mahidol University, Bangkok, Thailand

**Keywords:** manifestations, rheumatic carditis, valvular regurgitation, predictors, mitral regurgitation, rheumatic carditis, Thai children

## Abstract

**Background:**

Acute rheumatic fever (ARF) with carditis can lead to the development of rheumatic heart disease in children and young adults.

**Objective:**

This study aimed to investigate the manifestations of rheumatic carditis, clinically significant regression of valvular regurgitation as assessed by echocardiography, and the independent predictors of mitral regurgitation (MR) improvement after rheumatic carditis in Thai children.

**Method:**

Children diagnosed with rheumatic carditis during 2005–2020 at Siriraj Hospital (Bangkok, Thailand) were retrospectively enrolled. Trivial, and mild regurgitation were grouped as non-clinically significant (NCS) regurgitation. Valvular regression was defined moderate-severe regurgitation improving to NCS regurgitation.

**Results:**

Eighty-one patients (mean age: 10 years, range: 8–12 years) were included. At presentation, 59 (72.8%) patients had combined mitral regurgitation (MR) and aortic regurgitation (AR), 20 (24.6%) patients had MR alone, and 2 (2.4%) patients had AR alone. Concerning severity, 28 (34.6%) and 30 (37%) patients presented with severe and moderate MR, respectively. Severe and moderate AR was found in 9 (11.1%) and 16 (19.8%) patients, respectively. At the one-year follow-up, 43.4% of moderate-severe MR, and 41.7% of moderate-severe AR improved to NCS regurgitation. Multivariate analysis revealed high erythrocyte sedimentation rate (ESR) (*p* = 0.01) and severe carditis (*p* = 0.05) at presentation to be independent predictors of MR improvement.

**Conclusion:**

Thai children with rheumatic carditis had a high incidence of valvular regurgitation; however, the valvular damage was improved in most patients. High ESR and severe carditis independently predict MR improvement.

## Introduction

Acute rheumatic fever (ARF) is a multisystem inflammatory autoimmune disease that is caused by group A streptococcal (GAS) infection [1,2,3,4]. ARF varies greatly in severity and organ involvement. Although the other manifestations are self-limiting and resolve without sequelae, carditis may present with severity that can range from asymptomatic to severe. Rheumatic carditis may lead to rheumatic heart disease (RHD), which is a major cardiovascular problem in children and young adults [5,6,7,8,9]. The incidence of RHD after ARF varied among studies [6,10,11,12,13]. The factors associated with the natural progression of the disease have been reported. Among these factors, the initial severity of carditis was identified as a potential predictor of outcomes. Two Brazilian studies based on clinical data revealed that children diagnosed with moderate or severe carditis faced a significantly higher risk of developing severe RHD compared to those with mild or no carditis [14,15]. Similar to the report from Australia [16], half of severe RHD patients underwent valve surgery within two years. On the contrary, Turkish data [8] did not find the association between the valvular regurgitation persistence and the initial valvular regurgitation. The socioeconomic status and utilization of antibiotic prophylaxis were identified as significant factors associated with a poorer prognosis in ARF [7,14,17]. Fabi et al. compared Rwandan and Italian rheumatic carditis in children [7]. The more favorable evolution in Italian children was reported. While, the more severe cardiac involvement, the higher rate of valve surgery, cardiovascular complications, and deaths were reported in Rwandan patients who had a different socioeconomic setting and lower adherence to secondary prophylaxis. The REMEDY study also demonstrated significant association of lack of secondary prophylaxis with the occurrence of ARF, heart failure, or death [18]. The global burden of RHD, resulting from the consequences of acute rheumatic carditis, is particularly significant in low- and middle-income countries [5]. On the other hand, the improvement of the valvular regurgitation has been recognized in post-ARF patients but no previous study has investigated the significant/independent predictors of regression of valvular regurgitation.

Accordingly, the aim of this study was to investigate the disease regression and the independent predictors of valvular improvement after rheumatic carditis in Thai children.

## Methods

This chart review study retrospectively enrolled child and adolescents aged less than or equal to 18 years who were diagnosed with and treated for rheumatic carditis during 2005 to 2020 at the Division of Pediatric Cardiology of the Department of Pediatrics, Faculty of Medicine Siriraj Hospital, Mahidol University, Bangkok, Thailand. We included patients who had a first diagnosis of acute rheumatic fever with carditis based on the revised Jones criteria [19]. Patients who did not undergo echocardiography at initial diagnosis and/or during follow-up were excluded. Electronic medical records were reviewed and baseline characteristics, including biographical information, clinical presentation, and medical history were recorded. All patients underwent first echocardiographic examination within 24 to 48 hours after the diagnosis of acute rheumatic fever was established. All the available follow up echocardiographic data was recorded. All patients enrolled in this study underwent at least one follow-up echocardiographic study. The conclusion of the follow-up period was determined based on the latest echocardiographic examination conducted. The protocol for this study was approved by the Siriraj Institutional Review Board (SIRB) (COA no. Si 559/2021). Verbal assent and/or written informed consent to participate in this study was not obtained from study participants and/or their parents due to the retrospective nature of this study.

All patients were admitted and treated according to the Thai guideline for the diagnosis and management of acute rheumatic fever. The treatment protocol was determined according to the level of carditis severity. With significant cardiomegaly or heart failure, the moderate and severe carditis was define as moderate and severe valvular regurgitation, respectively [20]. A dose of 1–2 mg/kg/day of prednisolone was prescribed in patients with moderate to severe carditis for two to four weeks, which was then switched to high-dose aspirin (acetylsalicylic acid, ASA) for four to six weeks. Only high-dose aspirin was prescribed in patients diagnosed with mild carditis. Patients with moderate to severe carditis were admitted to the hospital for absolute bed rest for four to eight weeks. The duration of bed rest, prednisolone, and aspirin was guided by inflammatory markers. The compliance with ATB prophylaxis was reviewed on the medical record.

### Severity and Regression of Valvular Regurgitation

Two-dimensional (2-D) echocardiography and flow Doppler echocardiography were performed to evaluate cardiac chamber size and to assess the severity of valvular regurgitation and stenosis. The severity of mitral value regurgitation (MR) and aortic valve regurgitation (AR) was determined according to the Doppler echocardiographic criteria recommended by the American Society of Echocardiography [21]. Mild MR was defined based on criteria including normal left atrial (LA) and left ventricular (LV) size, as well as Doppler and quantitative measurements. All echocardiographic examinations were performed by pediatric cardiologists. To facilitate the comparison of left ventricular (LV) dimensions among children and adolescents of varying body sizes, z-scores were computed for LV end-diastolic and systolic dimensions. These z-scores were determined using body-surface area (BSA) normogram.(22)

The severity of MR and AR was classified as no, trivial, mild, moderate, or severe regurgitation. Trivial, and mild regurgitation were categorized as non-clinically significant regurgitation (NCS). Clinically significant (CS) regression of valvular regurgitation was defined as improvement from moderate-severe regurgitation to NCS regurgitation. During follow up, the first time of CS regression was used to analyzed. The outcome of interest in this study was the occurrence of regression from CS severity to NCS severity.

## Statistical Analysis

Descriptive statistics were used to summarize patient demographic and clinical characteristics. Normally distributed and non-normally distributed continuous data are presented as mean plus/minus standard deviation (SD) and median and interquartile range (IQR), respectively. Categorical data are expressed as number and percentage. Normally distributed and non-normally distributed continuous data were compared using Student’s *t*-test and Mann-Whitney U test, respectively. Chi-square testing was used to compare categorical data. The duration to first time of CS regression was performed using Kaplan-Meier survival analysis, and the log-rank test was used to compare survival curves. Variables with a *p*-value less than 0.1 from the univariate logistic regression analysis were included in the multivariate Cox regression model to identify independent predictors of improvement from moderate-severe mitral MR to non-clinically significant (NCS) MR. The results of the univariate and multivariate Cox regression analyses are reported as hazard ratio (HR) and 95% confidence interval (95%CI) and adjusted HR (aHR) and 95%CI, respectively. A *p*-value less than 0.05 was considered statistically significant for all tests. All statistical analyses were performed using PASW Statistics version 18 (SPSS, Inc., Chicago, IL, USA).

## Results

We retrospectively enrolled 81 patients with a first diagnosis of ARF with carditis. The median age at the initial presentation was 10.5 years (IQR: 7.7–13.3 years), and the median follow-up duration was 7.5 years (IQR: 4.3–13 years).

Concerning the major criteria for acute rheumatic fever at presentation, 20 (24.7%) patients had migratory arthritis, which was found in the knee in 17 (21.0%) patients, in the ankle in 17 (21.0%) patients, and in the wrist in 5 (6.2%) patients. Sydenham’s chorea was presented in 8 (9.9%) patients. Only 3 (3.7%) patients had subcutaneous nodules. Erythema marginatum was not detected in our study population. The minor criteria are shown in Table 1. During follow-up, 11 (13.6%) patients had recurrent ARF. The median time to recurrence of ARF from the index presentation was 1.97 years (range 0.16–5.11 years).

**Table 1:** Baseline Characteristics, Clinical Manifestations, Diagnostic Details, And treatment Among Overall Patients, And compared Between those Who Did and Did Not Develop Recurrent ARF During Follow-Up.


*PATIENT CHARACTERISTICS*	ALL PATIENTS (n = 81)	MITRAL VALVE REGRESSION DURING FOLLOW-UP (n = 46)	NO MITRAL VALVE REGRESSION DURING FOLLOW-UP (n = 35)	p-VALUE

Demographic/anthropometric data				

– Age at onset (years)	10.5 ± 2.8	9.5 ± 2.5	10.6 ± 2.9	0.07

– Male gender	45 (55.6%)	24 (52.2%)	21 (60.0%)	0.51

– Weight (kg)	30 (21.5, 43.2)	29.2 (20.8, 39.9)	31.8 (23.2, 51.5)	0.20

– Height (cm)	136.9 ± 17.9	133.6 ± 17	141.4 ± 18.3	0.05

– Body surface area (m2)	1.1 ± 0.3	1.1 ± 0.3	1.2 ± 0.3	0.09

Major manifestations				

– Migratory polyarthritis	20 (24.7%)	13 (28.3%)	7 (20%)	0.44

– Sydenham chorea	8 (9.9%)	4 (8.7%)	4 (11.4%)	0.72

– Subcutaneous nodule	3 (3.7%)	1 (2.2%)	2 (5.7%)	0.57

Minor manifestations				

– Fever	61 (75.3%)	37 (80.4%)	24 (68.6%)	0.30

– Arthralgia	29 (35.8%)	14 (30.4%)	15 (42.9%)	0.35

– Elevated ESR/CRP	75 (92.6%)	45 (97.8%)	30 (85.7%)	0.08

– Prolonged PR interval	14 (17.3%)	7 (15.2%)	7 (20%)	0.77

– ESR (mm/hr)	66 (42, 98)	76.5 (51.5, 102.2)	61 (32, 86)	0.03

– CRP (mg/L)	35.5 (13, 84.5)	29.7 (11.6, 82.2)	50 (16.9, 84.5)	0.39

Evidence of a preceding GAS infection				

– Positive throat swab culture (n = 57)	4 (7.0%)	3 (9.4%)	1 (4.0%)	0.62

– ASO titer (n = 78)				

– Positive	76 (97.4%)	43 (97.7%)	33 (97.1%)	1.00

– Value (IU/ml)	563 (355.5, 1090)	567 (400, 1004.5)	545 (310, 1220)	0.98

– Anti-DNaseB (n = 47)				

– Positive	43 (91.5%)	17 (81%)	26 (100%)	0.03

– Value (U/ml)	703 (402, 1089)	653.5 (323, 1097.5)	757.5 (435.7, 1089.2)	0.55

Treatment				

– Admission (days)	33.64 ± 24.40	53.3 ± 79	37.7 ± 44.8	0.23

– Bed rest (days)	30 (28, 60)	30 (28, 48.7)	30 (14, 60)	0.83

– Prednisone administration				

– Patients	66 (81.5%)	38 (82.6%)	28 (80%)	0.78

– Duration (months)	1.3 (0.9, 2.2)	1.3 (1, 1.9)	1.3 (0.9, 2.4)	0.88

– Dose (mg/kg/day)	2 (2, 2)	2 (2, 2)	2 (2, 2)	0.90

– Aspirin administration				

– Patients	72 (88.9%)	41 (50.6%)	31 (38.3%)	1.00

– Duration (months)	4.69 (2.6, 7.0)	4.8 (2.7, 6.9)	4.5 (2.4, 7)	0.73

– Dose (mg/kg/day)	80 (75, 84.7)	80 (75, 82)	80 (75, 85)	0.70

– Compliance with ATB prophylaxis	68 (84%)	40 (87%)	28 (80%)	0.54

Recurrent ARF				

– Recurrent ARF (n = 11)	11 (13.6%)	6 (13%)	5 (14.3%)	1.00


Data presented as mean ± standard deviation, median and interquartile range, or number and percentage. A *p*-value < 0.05 indicates statistical significance. **Abbreviations:** anti-DNaseB, antideoxyribonuclease B; ARF, acute rheumatic fever; ASA, acetylsalicylic acid or aspirin; ASO titer, anti-streptolysin O titer; ATB, antibiotic drugs; CRP, C-reactive protein; ESR, erythrocyte sedimentation rate; GAS infection, group a streptococcal infection; Prolonged PR interval, delayed conduction between the atria and the ventricles.

**Figure 1:**
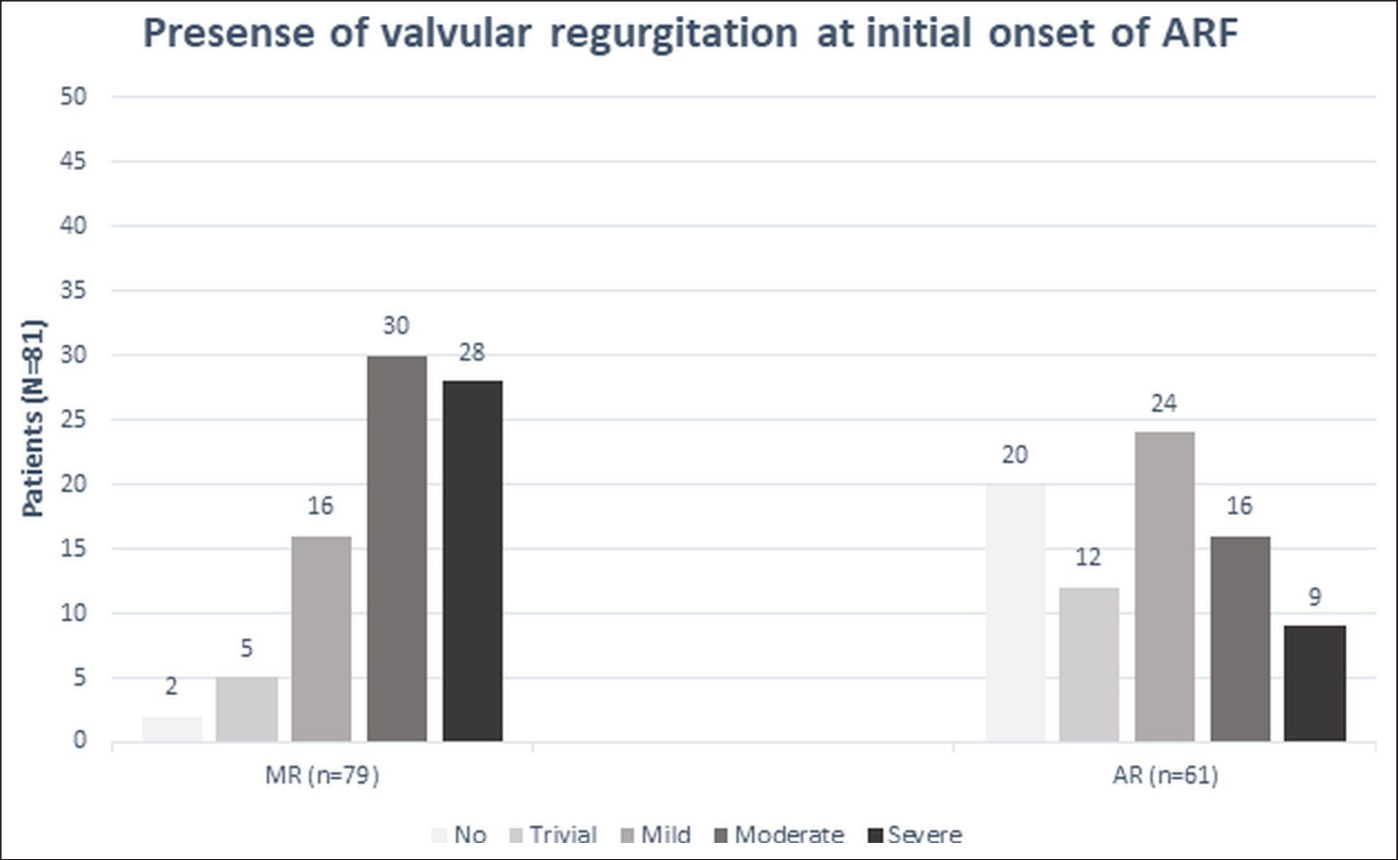
The incidence Of Mitral Regurgitation (MR) And/or Aortic Regurgitation (AR) At The Initial Onset of acute rheumatic fever (ARF) Stratified According To MR And AR Severity.

Cardiac manifestations included heart murmurs in 71 (87.7%) patients, heart failure in 48 (59.3%) patients, pericardial effusion in 14 (17.3%) patients, arrhythmias in 7 (8.6%) patients, and cardiogenic shock in 3 (3.7%) patients. Prolongation of the PR interval as a minor criterion was found in 14 (17.3%) patients. The echocardiographic findings specific to the left ventricular size and systolic function, right ventricular systolic pressure, and the presence of pericardial effusion are shown in Table 2. Among all patients, 59 (72.8%) patients had combined MR and AR at the initial onset of rheumatic carditis, whereas MR alone was presented in 20 (24.6%) patients and AR alone was presented in 2 (2.4%) patients. The incidence of MR and/or AR at the initial onset of ARF stratified according to MR and AR severity is shown in Figure 1. Moderate and severe carditis were found in 35 patients (43.2%) and 30 patients (37%), respectively. Of the 11 (13.6%) patients that required surgery, only one patient required surgery at the acute stage and the rest patients underwent surgery during the follow up. Seven (63.6%) patients underwent mitral valve repair/replacement and 4 (36.4%) patients underwent operation on both the mitral valve and the aortic valve. None of them required the re-intervention during the follow up.

**Table 2:** Echocardiographic findings at the initial onset of ARF among overall patients, and compared between those who did and did not develop recurrent ARF during follow-up.


FINDINGS	ALL PATIENTS (n = 81)	MITRAL VALVE REGRESSION DURING FOLLOW-UP (n = 46)	NO MITRAL VALVE REGRESSION DURING FOLLOW-UP (n = 35)	p-VALUE

At presentation				

Presence with MR alone	16 (19.8%)	7 (20.0%)	9 (19.6%)	0.96

Z-score-LVEDD	2.32 (0.70, 3.27)	2.91 (1.70, 3.65)	2.12 (–0.02, 2.84)	0.001

Z-score-LVESD	2.04 (0.78, 3.27)	2.69 (1.67, 3.45)	1.38 (0.19, 2.87)	0.002

LVEF (%)	63.3 ± 6.7	62.6 ± 7.8	63.43 ± 6.6	0.74

RVSP (mmHg)	42.56 (29.3, 57.3)	38 (32, 50)	44 (28.7, 59)	0.98

Pericardial effusion (%)	28 (34.6%)	5 (45.4%)	23 (32.9%)	0.50

At last follow up				

z-score-LVEDD	0.74 (–0.39, 2.64)	0.52 (–0.45, 1.93)	1.27 (–0.12, 2.88)	0.2

z-score-LVESD	0.87 (–0.13, 2.22)	0.63 (–0.15, 1.78)	1.18 (–0.12, 2.32)	0.64


Data presented as mean ± standard deviation, median and interquartile range, or number and percentage. A *p*-value < 0.05 indicates statistical significance. **Definitions:** ARF, acute rheumatic fever; LVEDD, left ventricular end-diastolic diameter; LVEF, left ventricular ejection fraction; LVESD, left ventricular end-systolic diameter; RVSP, right ventricular systolic pressure.

The mean duration of hospital admission was 33.6 ± 24.40 days. Prednisolone and aspirin were administrated in 66 (81.5%) and 72 (88.9%) patients, respectively. Three patients who received aspirin had major adverse effects, which included transaminitis in two patients and epistaxis that required treatment in one patient. The treatment-related details are shown in Table 1. As secondary prophylaxis, oral penicillin and benzathine penicillin were prescribed in 58.3% and 41.7% of all patients. The rate of compliance with antibiotic (ATB) prophylaxis was not significantly different those who did and did not develop recurrent ARF during follow-up (81.8% vs. 85.7%, respectively; *p* = 0.13).

### Changes of Mitral Valve and Aortic Valve Regurgitation

**Figure 2:**
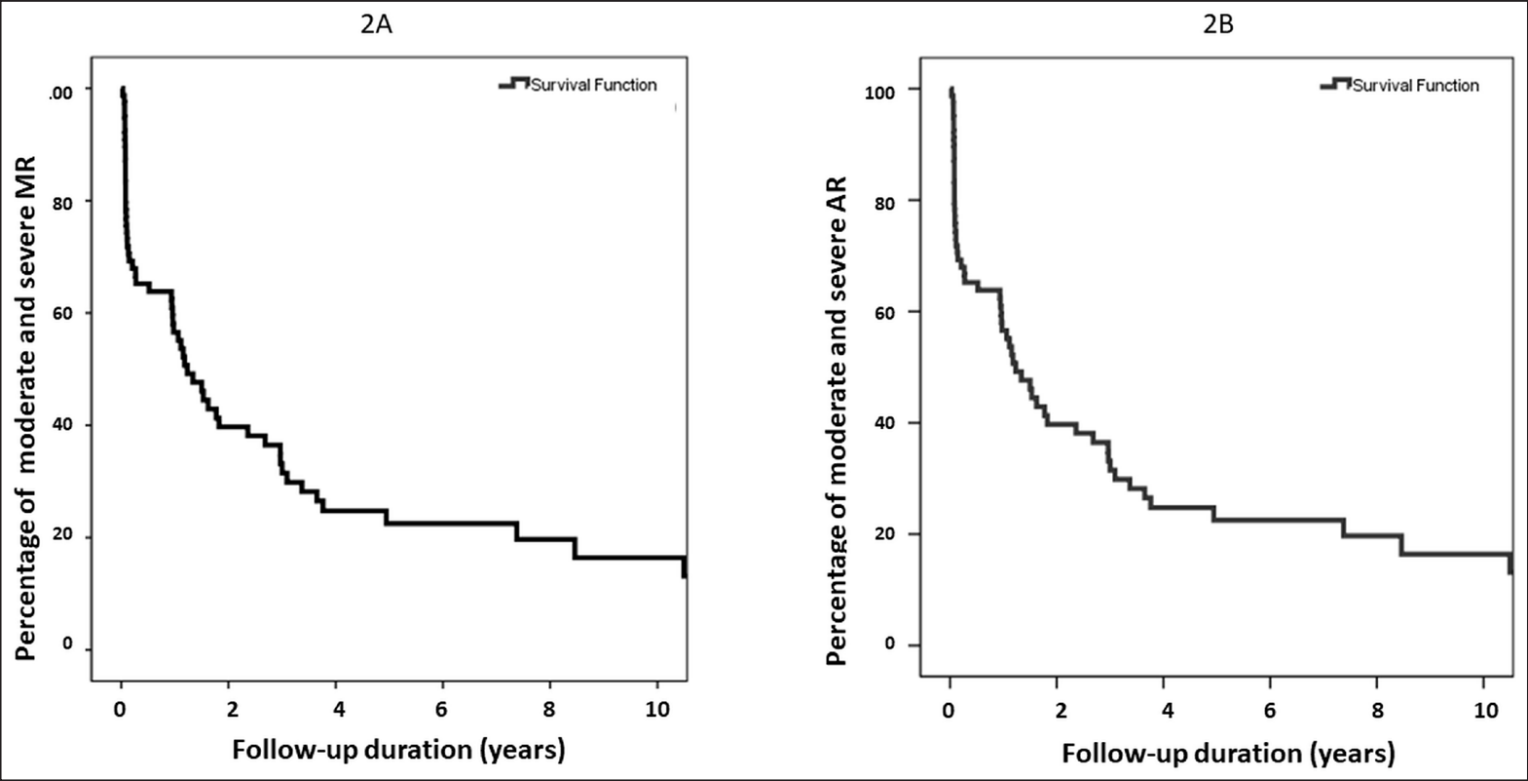
Kaplan-Meier Survival analysis Was used To Evaluate the Duration From **(A)** Moderate-Severe mitral regurgitation (mr) To Non-clinically significant (NCS) MR, And From **(B)** Moderate-Severe Aortic Regurgitation (AR) To NCS AR.

Regression of MR and AR severity was evaluated by echocardiography in this study. At the initial presentation, 28 (34.6%) and 30 (37.0%) patients presented with severe and moderate MR, respectively. Severe and moderate AR was found in 9 (11.1%) and 16 (19.8%) patients, respectively (Figure 1). During the follow-up period, the severity of MR and AR showed a trend toward regression to NCS regurgitation. Kaplan-Meier survival analysis was used to evaluate the duration from moderate-severe MR to NCS MR, and from moderate-severe AR to NCS AR (Figure 2). At the one-year follow-up, 43.4% of moderate and severe MR regressed to NCS MR, and 41.7% of moderate and severe AR regressed to NCS AR. On the other hand, 4.3% of initial NCS MR evolved to clinical significant MR and 3.6% of initial NCS AR evolved to clinical significant AR at one-year follow up.

### Predictors of Regression of MR

Cox regression univariate analysis showed higher erythrocyte sedimentation rate (ESR), presentation with severe carditis and larger z-scores of left ventricular end-diastolic diameter (LVEDD) and left ventricular end-systolic diameter (LVESD) to be significant predictors of regression to NCS MR. Multivariate analysis revealed high ESR and severe carditis at the initial presentation to be strongly independently associated with regression to NCS MR (Table 3).

**Table 3:** Cox proportional univariate and multivariate analysis to identify factors independently associated with clinically significant MR regression.


POTENTIAL PREDICTORS OF MITRAL VALVE REGRESSION	UNIVARIATE ANALYSIS		MULTIVARIATE ANALYSIS	
	
	HR (95%CI)	p*	AHR (95%CI)	p

Age at presentation	0.93 (0.83–1.04)	0.218		

Female gender	1.06 (0.59–1.91)	0.842		

Domicile in capital city	0.90 (0.47–1.72)	0.747		

ESR level	1.01 (1.00–1.02)	0.007	1.01 (1.01 – 1.03)	0.01

CRP level	1.00 (1.00–1.01)	0.798		

Presented with heart failure	1.35 (0.73–2.50)	0.332		

Severe carditis at presentation	5.83 (1.80–18.91)	0.003	4.14 (1.02–16.86)	0.05

Administration of glucocorticoids	0.99 (0.93–1.06)	0.759		

Duration of prednisolone treatment	0.99 (0.95–1.03)	0.590		

Duration of ASA treatment	1.00 (0.99–1.01)	0.741		

Duration of bed rest	1.00 (0.99–1.01)	0.741		

Good compliance with ATB prophylaxis	1.29 (0.54–3.04)	0.566		

Presence with MR alone	0.56 (0.26–1.20)	0.136		

Z-score LVEDD at presentation	1.39 (1.15 – 1.69)	0.001	1.12 (0.87 – 1.46)	0.381

Z-score LVESD at presentation	1.46 (1.16 – 1.83)	0.001		

LVEF	1.00 (0.95–1.06)	0.919		

Z-score LVEDD at last follow up	0.94 (0.80 – 1.10)	0.426		

Z-score LVESD at last follow up	0.97 (0.93 – 1.14)	0.727		

History of recurrent ARF	0.89 (0.38–2.11)	0.793		


A *p*-value < 0.05 is considered statistically significant. *Factors with a *p*-value < 0.20 from univariate logistic regression analysis were included in the multivariate analysis. **Abbreviations:** aHR, adjusted hazard ratio; AR, aortic valve regurgitation; ASA, acetylsalicylic acid or aspirin; ATB, antibiotic drugs; CI, confidence interval; CRP, C-reactive protein; ESR, erythrocyte sedimentation rate; HR, hazard ratio; LVEDD, left ventricular end-diastolic diameter; LVEF, left ventricular ejection fraction; LVESD, left ventricular end-systolic diameter; MR, mitral valve regurgitation.

## Discussion

Our study reports the clinical presentations/manifestation and outcomes of pediatric rheumatic carditis in Thailand. To our knowledge, this is the first study to investigate the predictors of regression of valvular regurgitation as evaluated by echocardiography in Thai children diagnosed with rheumatic carditis.

The prevalence of ARF/RHD in Thai children was reported was reported to have declined from 1.13 per 1,000 in 1986 to 0.23 per 1,000 in 2016 [23]. Another report described Thailand as being non-endemic for rheumatic heart disease [24]. However, there was no strong evidence of why the prevalence of ARF/RHD in Thai children has decreased. The potential reason may be the accessibility of medical treatment has been improved after the announcement of universal coverage for health insurance in 2001. Therefore, most children would get the appropriate treatment for Streptococcal infection. However, ARF and RHD still remain important causes of cardiovascular morbidity and mortality in Thailand and worldwide [15,25,26].

The recurrence rate of ARF observed during the follow-up in our study was lower than those reported from Malaysia and other Southeast Asian countries, but comparable with those reported from developed countries [11,27,28]. Regarding clinical presentation and diagnostic criteria, there was no significant difference between the recurrence and non-recurrence during follow-up groups in our study. Concerning ATB prophylaxis, intramuscular benzathine penicillin was reported to be superior to oral penicillin V for preventing a recurrence of ARF [29]. In contrast to the study from United States [30] in which most of their patients were prescribed benzathine penicillin, most of our patients received oral penicillin because benzathine penicillin is not always available in Thailand, and ATB compliance was good overall. However, the ATB compliance may be limited to interpret due to the nature of retrospective study. Despite our findings not revealing an association between ATB prophylaxis and the regression of valvular regurgitation, our study observed a relatively low recurrence rate of ARF during the follow-up period. Based on these results, it may be reasonable to consider oral penicillin with good compliance as a viable option for secondary prevention in settings with limited resources. It is well established that the mitral valve is the most affected valve in ARF [2,7,31]. In our study, MR with coexisting AR was the most commonly observed abnormality. Only 2.4% of patients in our study had AR alone. The sequelae of permanent valve damage lead to rheumatic heart disease (RHD). The revolution of valvular regurgitation has been published before and after availability of echocardiography. Previous studies reported that without specific treatment, 40–60% of valvular regurgitation regressed after the first episode of ARF [32,33,34]. Low socioeconomic status was also found to be associated with a lower rate of improvement of valvular lesions [7].

Narin et al. reported improvement of MR and AR in 51% and 20% of patients, respectively, at one year after the diagnosis [31]. Consistent with previous reports, our study observed regression of both MR and AR to NCS regurgitation in 43.4% and 41.7% of cases, respectively, at the one-year follow-up. This presented data elucidates the burden of disease and highlights potential of the favorable outcomes.

In addition to environmental and genetic factors, immune mechanisms following a GAS infection also play an important role in causing ARF and subsequent disease progression to RHD [35]. Both innate and adaptive immune mechanisms are activated, which causes endothelial injury. Extensive fibrosis, collagen deposition, and valvular scarring can be identified following the healing process [35]. Nevertheless, the understanding of how severity of inflammation impacts clinical outcomes remains limited. CRP and ESR are the most important markers of inflammation. Our study found a high ESR level at the initial presentation to be independently associated with regression of MR. The ESR may serve as a prognostic indicator of the outcomes in ARF. In our population, the corticosteroid was administrated in moderate and severe carditis. Based on our data, we are still not able to prove the benefit on the treatment with corticosteroid as it failed to be statistical significance on multivariate analysis. However, this is a retrospective study with nature of patient biases and limitation of the data’s accuracy and completeness as well as lack of the control group to demonstrate the efficacy of treatment strategies. Further study in the impact of anti-inflammatory treatments in this setting is warranted.

Similar to our results, most patients with rheumatic carditis were found to have normal left ventricular systolic function with left ventricular (LV) dilatation [15,36]. LV dilation was associated with the severity of valve involvement [8,15], but also occurred in the absence of hemodynamically significant valvular regurgitation [37]. In other words, a larger LV size may be the result of more severe valvular regurgitation and also represent myocardial involvement in severe carditis. The previous study [8], initial LV dilatation was identified as a factor associated with persistent valvular involvement. On the contrary, we found larger LV size associated with disease regression on cox univariate analysis but lost statistic power on cox multivariate analysis.

The natural history of patients diagnosed with rheumatic carditis varies in the severity of disease. Clinical data from Brazilian studies showed that children with moderate or severe carditis had a greater risk of developing severe RHD when compared to those who had mild or no carditis [14]. Another study demonstrated patients having a lower severity of carditis at the initial presentation were found to have a greater likelihood of murmur disappearance during the follow-up period [32]. The Australian study [16] showed equal chance of disease progression or regression in moderate RHD and the worse outcomes in patients with severe RHD. On the other hand, the study from Turkey did not find the significant correlation between initial severity of valvular involvement and chronic valvular disease [8]. In Australia, the operation was indicated in the children with severe RHD in New York Heart Association Class II. As a result, 41% of severe RHD underwent surgery within first year of the diagnosis. In contrast to our data, we observed an independent association between severe carditis and the regression of mitral regurgitation (MR) during our extended follow-up period. In our clinical approach, we adopted a more conservative stance towards performing surgery in patients with severe carditis. Surgical intervention was reserved for individuals experiencing severe heart failure that remained unresponsive to medical treatment. Consequently, we tended to delay the timing of surgery, potentially affording greater opportunities for valvular remodeling in our patients with severe carditis.

Our findings revealed that higher ESR and the presence of severe carditis were associated with disease regression. These factors indicate a more severe form of the disease. Although we did not establish the exact underlying patho-mechanism for these results, our study demonstrated the potential for valvular regression in patients with moderate and severe disease. Consequently, we recommend that patients with moderate or severe valvular regurgitation receive appropriate management, allowing for an adequate period of remodeling, as this is crucial for achieving favorable patient outcomes.

## Limitations

This study has some mentionable limitations. First, this was a single-center study. Second, there exists a risk of selection bias due to the fact that our center is a large university-based tertiary referral center that is routinely referred complex cases thought not to be treatable at other levels of care. As such, our findings may not be immediately generalizable to other levels of care. Third, our study had a retrospective design, which rendered it vulnerable to certain biases and missing/incomplete data in some cases. Fourth, our study’s retrospective design with relative small number made it difficult to evaluate and compare the efficacy of treatment and compliance with ATB prophylaxis. Fifth and last, there was a variation in the follow-up duration that resulted from differences in the follow-up protocols among pediatric cardiologists.

## Conclusion

ARF and RHD continue to be important causes of cardiovascular morbidity and mortality in Thailand. Thai children with rheumatic carditis had a high incidence of valvular regurgitation; however, the valvular damage improved during the follow-up period in most patients. High ESR and large LVEDD at the initial presentation were found to independently predict MR improvement. The future study on the efficacy of treatment strategies and the impact of level of inflammation is required for achieving the better outcomes in post ARF patients.

## Data Accessibility Statement

The datasets used and/or analyzed during the current study are available from the corresponding author on reasonable request.
